# An Area-Efficient and Highly Linear Reconfigurable Continuous-Time Filter for Biomedical Sensor Applications

**DOI:** 10.3390/s20072065

**Published:** 2020-04-07

**Authors:** Jinyong Zhang, Shing-Chow Chan, Hui Li, Nannan Zhang, Lei Wang

**Affiliations:** 1 College of Big Data and Internet, Shenzhen Technology University, Shenzhen 518118, China; zhangjinyong@sztu.edu.cn; 2 Department of Electrical and Electronic Engineering, The University of Hong Kong, Hong Kong 999077, Hong Kong; 3 Shenzhen Institutes of Advanced Technology, Chinese Academy of Sciences, Shenzhen 518055, China; 4 School of Science and Engineering, The Chinese University of Hong Kong, Shenzhen 518172, China; nnzhang@siat.ac.cn

**Keywords:** reconfigurable filter, continuous-time, low frequency, low-pass filter (LPF), notch filter (NF), current-steering (CS), biomedical sensors

## Abstract

This paper proposes a compact, high-linearity, and reconfigurable continuous-time filter with a wide frequency-tuning capability for biopotential conditioning. It uses an active filter topology and a new operational-transconductance-amplifier (OTA)-based current-steering (CS) integrator. Consequently, a large time constant τ, good linearity, and linear bandwidth tuning could be achieved in the presented filter with a small silicon area. The proposed filter has a reconfigurable structure that can be operated as a low-pass filter (LPF) or a notch filter (NF) for different purposes. Based on the novel topology, the filter can be readily implemented monolithically and a prototype circuit was fabricated in the 0.18 μm standard complementary-metal–oxide–semiconductor (CMOS) process. It occupied a small area of 0.068 mm^2^ and consumed 25 μW from a 1.8 V supply. Measurement results show that the cutoff frequency of the LPF could be linearly tuned from 0.05 Hz to 300 Hz and the total-harmonic-distortion (THD) was less than −76 dB for a 2 Hz, 200 mVpp sine input. The input-referred noises were 5.5 μVrms and 6.4 μVrms for the LPF and NF, respectively. A comparison with conventional designs reveals that the proposed design achieved the lowest harmonic distortion and smallest on-chip capacitor. Moreover, its ultra-low cutoff frequency and relatively linear frequency tuning capability make it an attractive solution as an analog front-end for biopotential acquisitions.

## 1. Introduction

Global population aging produces a strong demand for portable and wearable biomedical sensor devices for the continuous monitoring of physiological signals in preventive and personalized healthcare. Ideally, these devices should possess a high precision, low power consumption, and small size. The performance of such biopotential signal acquisition systems depends critically on the analog front-end (AFE) [1]. Analog filters are often preferred over digital filtering in the AFEs for their low power consumption, especially for multi-channel systems. Moreover, as most AFEs often support DC offset suppression through the use of chopper stabilization, which requires digital clocks, considerable interference is introduced. Therefore, band-limiting analog filters are often utilized to remove out-of-band noise (e.g., from clock signals or other interference). Such filters also provide anti-aliasing for subsequent A/D conversion and are essential for the removal of high-frequency noise, such as crosstalk from clock signals, power-line interference (PLI), etc.

In general, biopotential signals are characterized by their low amplitude (e.g., tens of microvolts to several millivolts) and significant low-frequency components (several millihertz to several hundreds of hertz) [2]. For example, the T-wave of an electrocardiograph (ECG) has its spectral content mostly centered around 2.4 Hz, for which the signal acquisition is often designed to have a cutoff frequency of about 150 Hz to ensure that all important information is captured [3,4]. Generally, the magnitude of ECG signals is from 1 mV to 5 mV, and the preamplifier is designed to have a moderate gain (e.g., 10) to avoid saturation of the amplifier due to the DC offset. Therefore, the input signal swing of the filter is in the order of tens of millivolts (e.g., 10–50 mV). Apart from area and power consumption considerations, as the biopotential is a delicate signal, attention should also be given to noise and linearity. 

The 50/60 Hz PLI is another significant and commonly encountered interference source when recording biopotential signals that is ubiquitous, even in clinical settings [5]. Noise sources can also originate from the surrounding environment, such as the patient’s residence, where many sources of ambient interference may arise. It is therefore desirable for the analog filters to be able to reconfigure either as a low-pass filter (LPF) or a notch filter (NF), or a combination of them with a wide tunable frequency characteristic, depending on the practical applications.

Conventionally, achieving a low cutoff frequency in a monolithic implementation for a continuous-time filter has always been a challenge as passive components are often large. The issue is even more serious for multi-channel systems, such as brain–machine interfaces (BMI), where power and area budgets are usually stringent. Much effort has been devoted to reducing the values of passive components while maintaining a good linearity. Operational-transconductance-amplifiers and capacitors (OTA-C)are usually preferred due to their simple and tunable structure [6,7]. However, they rely on current division mirrors to obtain extremely small *g_m_* values. As transistors biased in weak inversion can lead to a decreased matching of currents in current mirrors, degradation of the total-harmonic-distortion (THD)will occur as a result [3]. A switched-capacitor (SC) filter is another popular technology for realizing NFs and LPFs with a low cutoff frequency due to its low sensitivity and high precision [8,9]. However, extra clock generators and large capacitor ratios are required to implement a large time constant. Moreover, dynamic power consumption is large and its linearity performance is limited by metal–oxide–semiconductor (MOS) switch charge injection and clock feed-through. Biasing the MOS transistor in the subthreshold regime to form large pseudo-resistors is an effective technique for obtaining a very low and tunable *g_m_* [10,11]. However, most designs employing this scheme produce low linearity and large distortions. Floating-gate MOS transistors have been proven to be effective in realizing low cutoff frequency filters [12]. The main drawback is that a special double-poly process, which is often unavailable in standard -CMOS technologies, is involved [13]. From the above discussion, it is apparent that a reconfigurable filter structure with a good solution to achieve low cutoff frequency, small silicon area, and low signal distortion is highly desirable.

In this paper, we present an area-efficient, high-linearity, and reconfigurable second-order continuous-time filter architecture by exploiting current-steering (CS) integrators for biopotential recording sensors. It relies on an active filter topology to achieve a high linearity and a new realization of an OTA-based CS integrator to achieve an ultra-low cutoff frequency. Moreover, a new analog inverter was implemented in the proposed filter by adopting the non-linear pseudo-resistors compensation. The new integrator was based on an improved realization of the current-steering (α block) and a high-gain OTA with lowinput-referred noise. It achieved a large time constant τ and good linearity, while requiring a small silicon area. The improved α block avoided the RC network in the conventional α block in Moon and Song [14] and led to lower power consumption and a smaller silicon area. Compared to the α block approach in Wong et al. [15], the proposed integrator was incorporated into a new biquad reconfigurable filter with a stable closed-loop feedback such that high linearity and linear bandwidth tuning could be achieved with a small area and low power consumption. The noise and linearity of the proposed circuits were also thoroughly analyzed.

The proposed filter can be readily implemented monolithically using standard CMOS technology, achieving good linearity, a wide frequency-tuning range, and a small silicon area. Comparison with conventional designs [6,16,17] revealed that the proposed design achieved the lowest harmonic distortion and smallest on-chip capacitor among these designs. Furthermore, the proposed filter has a reconfigurable topology, which can be used as an LPF or an NF. 

The rest of this paper is organized as follows: Section 2 gives an overview of the proposed filter architecture and the detailed circuit implementation is described in Section 3. The analysis of the linearity, noise, and tuning performance of the proposed structure is presented in Section 4. The measurement results and comparisons are presented in Section 5. Finally, Section 6 concludes the paper. 

## 2. System and Architecture

**Figure 1:**
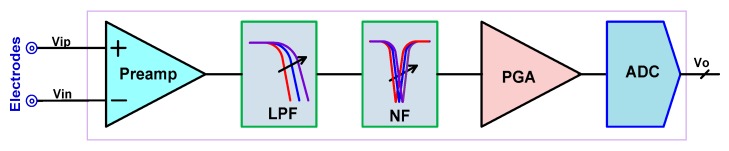
An example block diagram of an analog front-end (AFE) system for bio-potential recording.

Figure 1 shows the system block diagram of a typical AFE for biopotential recording. It consists of a preamplifier (Preamp), a bandwidth configurable low-pass filter (LPF) and a tunable notch filter (NF), a programmable gain amplifier (PGA), and an analog-to-digital converter (ADC).

As mentioned earlier, the main design challenges for the monolithic realization of such signal conditioning filters for biopotential acquisition are the requirement of a low cutoff frequency and linearity under given area and/or power limitations. Generally, active filters are more attractive for its smaller silicon area, but its linearity is determined by the voltage coefficient of the passive components only when the gain of the amplifier is high enough. Due to various nonlinear and imperfect properties of the circuit elements, special care is required to achieve a low cutoff frequency. Several techniques have been proposed to enhance the linearity in these low cutoff frequency filters, which include input attenuation, source degeneration, non-linear term cancellation, etc. However, most of these techniques depend on either a special CMOS process (e.g., a floating-gate MOS) or the accurate matching of MOS transistors [18]. Therefore, there is a recent trend in employing the classic approach [19] with high-gain active circuits in feedback loops and passive components to achieve a high linearity.

**Figure 2:**
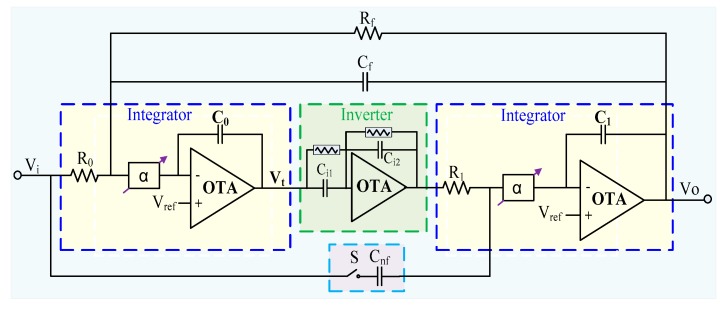
Block diagram and key features of the proposed reconfigurable filter based on the current-steering scheme.

In this work, a new reconfigurable second-order biquad filter structure was proposed to meet the high linearity and low cutoff frequency requirements for biopotential acquisitions. The proposed topology can be viewed as the integration of a traditional active filter structure with a new CS integrator, and it offers a good tradeoff between linearity and low-frequency bandwidth. Figure 2 shows the block diagram of the proposed second-order reconfigurable filter, which comprises two identical integrators, an analog inverter, and an RC feedback network. Each integrator utilizes an improved current-steering technique (α block) to provide a large time constant, a wide linear tuning range, and a low noise level comparable to the use of a conventional MOS pseudo-resistor.

The concept of an *α* block was first proposed by Moon and Song [14] for implementing a variable resistor for a Bessel filter and by Wong et al. [15] to realize a simple first-order LPF. As mentioned earlier, the new integrator was based on an improved realization of the α block and a new high gain OTA with lowinput-referred-noise. It achieved a large time constant τ and a good linearity, while requiring a small silicon area. It also avoids the need for an RC network in the conventional α block in Moon and Song [14], and in this way, leads to a lower power consumption and smaller silicon area. Compared to the α block in Wong et al. [15], the proposed *α* block and high gain OTA-based integrator were incorporated into a new biquad reconfigurable filter with a stable closed-loop feedback such that high linearity and linear bandwidth tuning could be achieved with a small area and low power consumption. More details are given later in Section 3. 

In the proposed structure, the NF or LPF operations can be configured depending on whether the CMOS switch S is ON or OFF. In practical applications, the switch is either ON or OFF when the working mode is chosen (i.e., LPF or NF). The switch consists of two complementary n-type MOS (NMOS) and p-type MOS (PMOS) with a small resistance that are located on the feedback loop (not the main signal path), and hence its effect on the system linearity is small. This provides considerable flexibility in practical applications where PLI and other high-frequency interference can be mitigated.

Using the α block in the integrator and the switch, the bandwidth of the filter can be tuned and reconfigured, respectively. To see this, let both positive terminals of the integrator be biased at $V_{ref}$($V_{ref}=\left(V_{DD}+V_{SS}\right)/2$), which give the filter a suitable dynamic range. The transfer function of the integrator employing the α block is then given by H(s)=1/(τs/α), where the parameter α is a scaling factor derived in Section 3. Thus, the complete continuous-time transfer function of the proposed filter described in Figure 2 is given by:

(1) $$H_{NF}(s)=\frac{1+\frac{R_{0}C_{0}R_{1}C_{nf}s^{2}}{\alpha^{2}}}{\frac{R_{0}}{R_{f}}{+R}_{0}C_{f}s+\frac{R_{0}C_{0}R_{1}C_{1}s^{2}}{\alpha^{2}}}.$$

If the resistances and capacitances are chosen such that $R_{f}{\;=R}_{1}{=R}_{0}$ and $C_{nf}{=C}_{f}{=C}_{1}{=C}_{0}$, Equation (1) can be used to realize an NF. The corresponding pass-band gain amplitude is close to unity and the central frequency of the NF is:

(2) $$f_{c,NF}=\frac{\alpha }{{2\pi R}_{0}C_{0}}.$$

The above equation shows that the central frequency of the *NF* is determined by the parameter α given the values of $R_{0}$ and $C_{0}$, and hence it can be controlled through α to achieve a specific frequency in practical applications.

As illustrated in Figure 2, when the switch S is set to OFF, the forward feedback capacitance $C_{nf}$ in Equation (1) is equal to zero, and the transfer function becomes:

(3) $$H_{LPF}(s)=\frac{1}{\frac{R_{0}}{R_{f}}{+R}_{0}C_{f}s+\frac{R_{0}C_{0}R_{1}C_{1}s^{2}}{\alpha^{2}}}.$$

Equation (3) is a classical transfer function of a biquad LPF when $R_{f}{=R}_{1}{=R}_{0}$ and $C_{nf}{=C}_{1}{=C}_{0}$ and its −3 dB frequency is given by:

(4) $$f_{c,LPF}=\frac{\alpha }{{2\pi R}_{0}C_{0}}.$$

The loop gain of the filter is defined by $R_{f}/R_{0}$ and it is set to one in the proposed design. One can see that, if α is set to a value much smaller than one, the cutoff frequency of the LPF can be lowered to the Hz or mHz range. The ultra-low cutoff frequency LPF is generally required for some vital signals, e.g., the slow varying photoplethysmogram (PPG) [15]. Thus, bio-signals with a very low frequency can also be processed by the proposed design. As the values of $R_{0}$ and $C_{0}$ are usually only several hundreds of ohm and several pF, respectively, the proposed reconfigurable filter can be readily implemented on-chip, even for multi-channel biopotential recording applications where the area per channel is limited. In principle, the linearity will be even better if a fully differential architecture is employed. However, it will consume a much larger area with more transistors and passive components. More importantly, as the α block is rather sensitive to the process, voltage, and temperature (PVT) variations, it is very difficult to handle the matching problem of the various components in the fully-differential structure. Therefore, we only focus on a single-ended design. Fortunately, from the comparison to be presented in Section 5, its impressive performance can still meet the demanding requirements in most practical applications. The detailed circuit implementation of various components of the proposed filter are shown in the following section.

## 3. Circuit Implementation

In this section, the basic principle of the proposed integrator and the circuit-level implementation of the filter are presented. The various design techniques used to satisfy the aforementioned design requirements of the reconfigurable biopotential filter are also highlighted. 

### 3.1. Current-Steering Integrator with an Improved α Block

**Figure 3:**
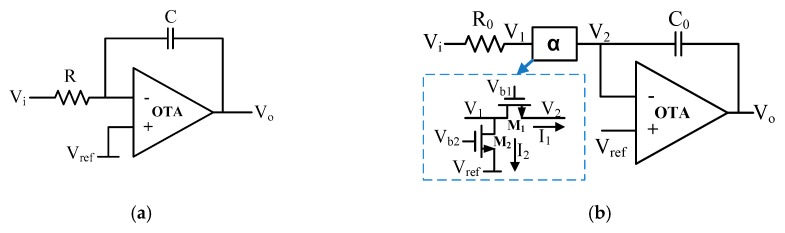
(**a**) The topology of a traditional RC integrator and (**b**) the proposed current-steering integrator and α block.

Integrators are critical building blocks for continuous-time filters as they determine both the frequency characteristic and the linearity. For designing filters with a low cutoff frequency, it is a great challenge to achieve a large integrator time constant while maintaining a high linearity. Due to the inherent nonlinearity of large MOS pseudo-resistors, the linearity of such filters reported thus far is limited to 40–50 dB [20,21]. Figure 3a shows the structure of a conventional first-order RC filter with a transfer function given by 1/(τs), where τ=RC is the time constant of the integrator. Here, a single-ended topology is shown for simplicity. The virtual ground of the amplifier allows for a highly linear voltage-to-current conversion, and the resistor current is then directly conveyed to the integrating capacitor. Thus, high-linearity is preserved. 

As mentioned, realizing a large time constant while keeping the values of the on-chip resistances and capacitances small for monolithic implementation is a big design challenge. To address this issue, an improved CS integrator and a high-gain OTA was proposed to obtain a large time constant τ and a low distortion. In the conventional α block [14], a complicated RC network is used to achieve a low distortion of approximately −90 dB, which leads to large power consumption and silicon area. The concept of an α block was also studied in Wong et al. [15], in which a second-order tunable low-pass filter was proposed. In this design, a cutoff frequency as low as 0.25 Hz was achieved by cascading two simple integrators. However, due to its single integrator architecture, its gain will change when the bandwidth is tuned by the gate voltage. Moreover, the linearity is limited by the simple integrator structure.

The proposed α block is based on a MOS-transistor-based current-sharing block. This is then incorporated in a high-gain OTA to form a novel CS integrator with improved linearity and a reduced gain variation during bandwidth tuning. Moreover, the improved CS integrator is incorporated into a new biquad reconfigurable filter with stable closed-loop feedback to achieve high linearity and a linear tuning bandwidth.

Figure 3b shows the CS integrator circuit and the details of the MOS-transistor-based *α* block. The proposed integrator architecture differs from the LPF of Wong et al. [15] in that it removes the feedback resistor. This is because the current through the *α* block is relatively small and hence the voltage $V_{1}$ is almost equal to the virtual ground. As a result, distortion and gain variation during bandwidth tuning are also reduced. Additionally, a high-gain OTA is employed to boost the linearity and lower the noise of the integrator. As illustrated in Figure 3b, the α block consists of two MOS transistors, which serve as current-sharing devices, biased in the deep triode region. 

The passive resistors and MOS transistors in the α block together create a variable resistance element with large values. To see that, let the current through *R*_0_ and the drain current of *M*_1_ and *M*_2_ be denoted by *I*_0_, *I*_1_, and *I*_2_, respectively. If *I*_2_ is much larger than *I*_1_, only a small portion of *I*_0_ will pass through *M*_1_ for charging or discharging the capacitor *C*_0_. Therefore, the filter time constant τ is increased significantly. Assuming $I_{1}/I_{0}=\alpha$, the transfer function of the CS integrator is given by:

(5) $$H(s)=\frac{1}{\tau (s/\alpha )},$$

where ${\tau =R}_{0}C_{0}$. It can be seen that the time constant τ of the proposed integrator is now increased by 1/α times comparing with the conventional one in Figure 3a. Moreover, if α≪1, the −3 dB frequency can be reduced significantly by a factor of α. 

The drain currents of *M*_1_ and *M*_2_, which are both biased in the linear region [22], can be expressed as:

(6a) $$I_{1}=\frac{1}{2}\mu_{n}C_{ox}{\left(\frac{W}{L}\right)}_{1}{(V}_{bi}\;{-\;V}_{ref}\;{-\;V}_{thn})\cdot {(V}_{1}\;{-\;V}_{2}),$$

(6b) $$I_{2}=\frac{1}{2}\mu_{n}C_{ox}{\left(\frac{W}{L}\right)}_{2}{(V}_{bi}\;{-\;V}_{ref}\;{-\;V}_{thn})\cdot {(V}_{1}\;{-\;V}_{ref}),$$

where $\mu_{n}$ is the electron mobility, *C_ox_* is the oxide capacitance, ${\left(W/L\right)}_{i}$ is the gate width-to-length ratio of *M_i_*, and $V_{thn}$ is the NMOS threshold voltage. 

When $I_{2}$ is much larger than $I_{1}$, the parameter α is approximately given by:

(7) $$\alpha \;=\frac{I_{1}}{I_{1}{+I}_{2}}\;\approx \;\frac{I_{1}}{I_{2}}\;\approx \;\frac{{(W/L)}_{1}{(V}_{b1}\;{-\;V}_{ref}\;{-\;V}_{thn})}{{(W/L)}_{2}{(V}_{b2}\;{-\;V}_{ref}\;{-\;V}_{thn})}.$$

**Figure 4:**
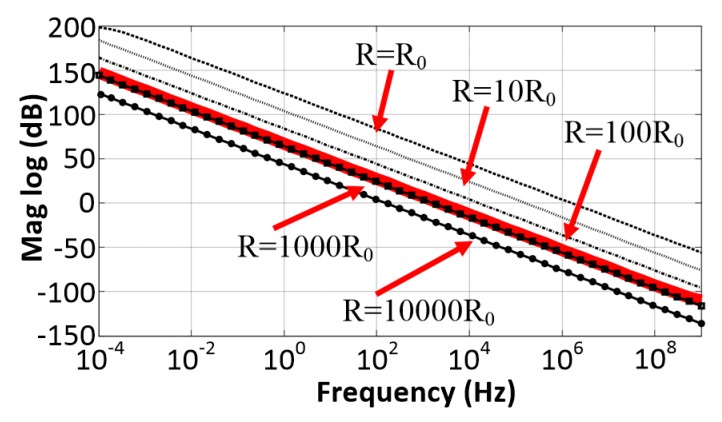
The AC responses of an RC integrator with different values of *R* (black lines) and the current-steering integrator (red line) with *R*_0_ = 100 kΩ.

Equation (7) indicates that the value of *α* can be tuned using the bias voltages *V_b_*_1_ and *V_b_*_2_. In practical implementation, when *M*_1_ steps into the subthreshold regime with a small *V_b_*_1_, a similar relationship between *α* and the bias voltages *V_b_*_1_ and *V_b_*_2_ can be obtained. Figure 4 shows the AC responses of the RC integrator with different values of *R* in Figure 3a and the proposed CS integrator in Figure 3b. In this simulation, the value of *R*_0_ in the proposed integrator was set to 100 kΩ and the value of *R* in the conventional integrator was chosen as a multiple of *R*_0_. It can be seen that the CS integrator could achieve similar linear gain–frequency responses and time constant τ as the conventional RC integrator, but at a much smaller resistance ($R_{0}=R/1000$).

### 3.2. Proposed OTA with High-Gain and Low-Input-Referred Noise

**Figure 5:**
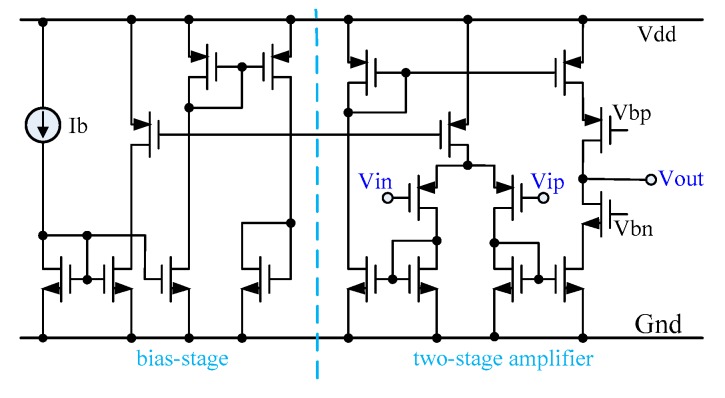
Transistor-level circuit of the OTA adopted in the current-steering (CS) integrator.

Figure 5 shows the transistor-level circuit of the OTA, which consists of the biasing circuit and a two-stage amplifier. The key design considerations are highlighted as follows: i) special attention was given to the design and layout of the input PMOS transistors to lower the flicker noise as it is significant at low operating frequencies [22] and ii) to effectively boost the OTA gain, NMOS and PMOS transistors were cascaded at the output stage to enhance the equivalent output resistance. This not only improved the integrator linearity but also lowered the OTA input-referred noise. Although the output dynamic range was somewhat lowered due to the large output resistance, it did not affect the performance of the OTA in our application. This was because the input swing of the OTA was small since the gain of the preamplifier was usually small for biopotential signals to avoid saturation by a large DC offset. Moreover, the gain of the filter was unity, and hence the input and output swings of the OTA were small, which was usually in the order of tens of millivolts.

### 3.3. Unity-Gain Analog Inverter with a Linearity Enhancement

The analog inverter is another block that can affect the attenuation of the proposed filter due to the non-linear pseudo-resistor in the feedback network. To quantify this effect, we assumed the input and output voltages were related using the following linear model:

(8) $$V_{io}{=-kV}_{ii}+∆V_{i},$$

where $V_{ii}$ and $V_{io}$ are the input and output of the unity-gain inverter, *k* is the proportionality constant between ${\;V}_{ii}$ and $V_{io}$, and $∆V_{I}$ is the offset voltage. Taking the NF as an example, the resultant transfer function in Equation (1) becomes:

(9) $$H_{NF}(s)=\frac{1+\frac{R_{0}^{2}C_{0}^{2}s^{2}}{{k\alpha }^{2}}-R_{0}C_{0}s\cdot \frac{∆V_{i}}{V_{ii}}}{{1+R}_{0}C_{0}s+\frac{R_{0}^{2}C_{0}^{2}s^{2}}{{k\alpha }^{2}}}.$$

**Figure 6:**
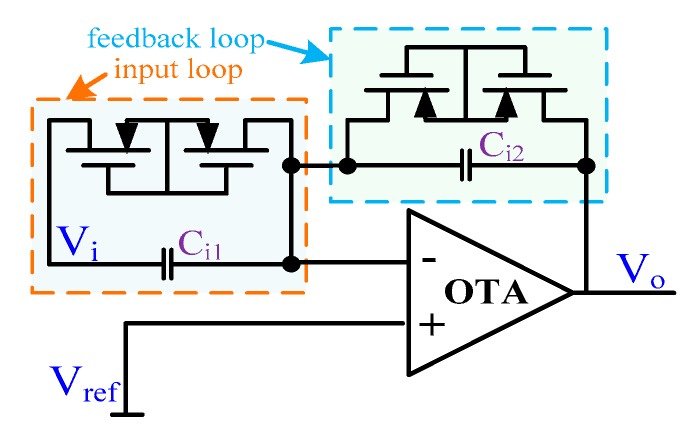
The topology of the unity-gain analog inverter.

Equation (9) indicates that the performance of the unity-gain analog inverter not only affects the cutoff frequency, but also the notch attenuation of the NF. The proposed linearity-enhanced unity-gain analog inverter is shown in Figure 6. This configuration introduces a pole as well as a zero for a pole–zero cancellation through the pseudo-resistor and capacitor in both the input loop and feedback loop, instead of only a pole in the feedback loop in the conventional design.

It is well-known that many low-frequency amplifiers and filters with pseudo-resistors in the feedback path have a limited linearity performance due to the inherent nonlinear behavior of MOS transistors in the triode regime. However, in this work, through careful design and optimization (e.g., a symmetrical layout design and a large aspect ratio of MOS transistors), the pole could be canceled by the zero in the proposed analog inverter to form an approximately ideal analog inverter. Furthermore, the equivalent resistances of the MOS-based pseudo-resistors in the input loop and feedback loop change with the input signal simultaneously. Through the symmetrical and unity-gain structure, the non-linear behavior of pseudo-resistors in this inverter is compensated for and thus the linearity of the inverter is enhanced. Cadence Spectre [23] simulation results showed that the designed analog inverter could achieve a −120 dB THD when the DC voltage of the input signal was biased at the middle of the power supply voltage ($\frac{V_{DD\;}{+V}_{SS}}{2}$). Consequently, the linearity issue in a conventional analog inverter can be significantly mitigated using the proposed methodology. 

## 4. Circuit Analysis

The performance of the proposed filter in terms of linearity, open loop-gain of the OTA, noise performance, and frequency tuning will be is discussed in this section. Key design considerations and techniques for achieving the desired performance metrics are also highlighted.

### 4.1. Linearity Performance

The main non-linear elements of the circuit are the MOS transistors in the *α* blocks, which operate as voltage control variable resistors. Traditionally, MOS-based resistors are usually applied in the feedback loop, where the voltage swing at the output is large, while the amplitude of the source signal at the input is relatively small. Thus, the use of MOS-based resistors in the feedback network exhibits nonlinear behavior, leading to a high-pass frequency point change and a large signal distortion in the analog chain [24]. In the proposed design, the MOS transistor *M*_1_ was designed to have better linearity than the conventional ones. From Equation (7) and Figure 3b, it is noted that the bypassing current ($I_{1}$) through *M*_1_ is scaled to $\alpha I_{2}$, resulting in the scaling down of *V*_1_ (see Figure 3b) since the current through the resistor *R*_0_ is unchanged. As a result of this scaling in *V*_1_, the signal swing across *M*_1_ decreases, and as such, the linearity improves. Although the input signal swing is slightly reduced, this linearity improvement is proportional to the amount of the scaling in *V*_1_.

In the proposed filter, the *α* blocks are located inside the feedback loop. This configuration can effectively reduce the distortion within the bandwidth of the filter. An analysis of the effects of current-steering MOS resistors in the feedback loop on filter performance can be found in Moon and Song [14]. 

The linearity of the proposed filter can be measured using the total harmonic distortion (THD) [25], which can be modeled using the following equation:

(10) $$\mathrm{THD}=\sqrt{\frac{\sum_{n=2}^{N}{\left|V_{out}{(nf}_{0})\right|}^{2}}{{\left|V_{out}{(f}_{0})\right|}^{2}}},$$

where $f_{0}$ is the fundamental frequency component, and the (*N*−1)th order harmonics are considered in the above formula. For this work, as shown in Figure 2, the filter has a linear feedback loop in which the even-order harmonics dominate. In fact, the second-order harmonic distortion (HD_2_) dominates, and thus the overall THD of the proposed filter can be approximated using HD_2_. The other reason for this approximation is that higher-order harmonics often lie beyond the filter stop-band and are thus filtered out. With the improved design and implementation of the proposed filter, CS integrator, OTA, and linear unity-gain analog inverter, as well as the biquad structure, the THD performance is significantly enhanced.

### 4.2. The Open-Loop Gain of the OTA

For an ideal active integrator, the OTA has an infinite open-loop gain and the dominant pole of the integrator lies at the zero frequency (e.g., for a DC). However, there are no other poles and finite-frequency zeros. The transfer function of an ideal non-inverting integrator is thus given by:

(11) $$H_{ideal}(s)=\frac{\omega_{0}}{s}.$$

**Figure 7:**
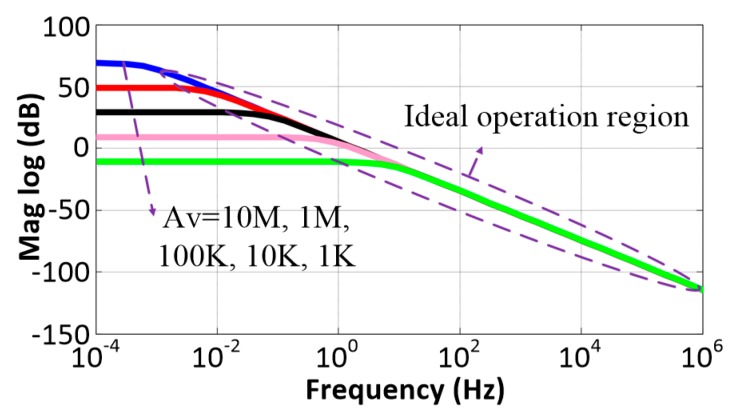
Relationship between the open-loop gain change of the OTA and the cutoff frequency of the integrator.

**Figure 8:**
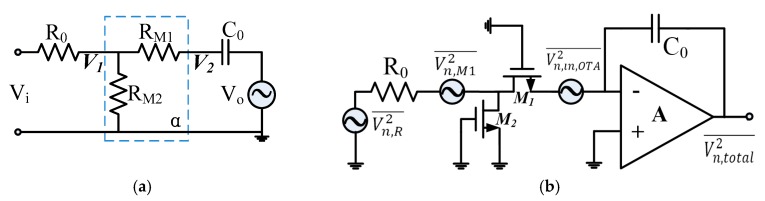
The small-signal equivalent model (**a**) and equivalent noise model (**b**) of the current-steering integrator.

The ideal characteristics of the integrator are illustrated in Figure 7 with a dashed line. In practice, the finite gain of the OTA will degrade the linearity of the active integrator. To analyze the non-ideal effect of the CS integrator, we denote the DC gain of the OTA by $A_{v}$, and the equivalent resistances of $M_{1}$ and $M_{2}$ by $R_{M1}$ and $R_{M2}$, respectively. From the small-signal equivalent model of the proposed integrator shown in Figure 8a, the transfer function was determined to be:

(12) $$H\prime (s)=-\frac{R_{0}{//R}_{M1}{//R}_{M2}}{R_{M1}C_{0}s+\frac{1}{A_{v}}\;-\;\frac{R_{0}{//R}_{M1}{//R}_{M2}}{A_{v}R_{M1}}}.$$

If the bias voltages $V_{b1}$ and $V_{b2}$ are appropriately chosen, and assuming that $R_{M1}\gg R_{M2}$ and $R_{0}\gg R_{M2}$, then Equation (5) can be rewritten as:

(13) $$H\prime (s)\;\approx \;-\frac{1}{\frac{R_{M1}R_{0}C_{0}s}{R_{M2}}\;-\;\frac{R_{0}}{A_{v}R_{M2}}},$$

which suggests that the CS integrator forms an active LPF with a low-frequency dominant pole at the frequency $\omega_{p}{=\omega }_{0}/A_{v}$. The real magnitude response with different DC gains of the OTA is shown by the solid lines in Figure 7, which indicates that the DC gain has a large impact on the CS integrator, i.e., better linearity and lower cutoff frequency can be achieved with a larger DC gain. Therefore, care should be taken in choosing the OTA DC gain and aspect ratio of MOS transistors to ensure that the transistors do not operate in their nonlinear region.

### 4.3. Noise Performance

To analyze the influence of noise, the equivalent noise model of the CS integrator given in Figure 8b was used. The equivalent noise power of transistor *M*_1_ at a low frequency is given by:

(14) $$\frac{\bar{V_{n,\;M1}^{2}}}{∆f}=4KT\frac{2}{3}R_{M1}+\frac{K_{f}}{C_{ox}W_{1}L_{1}}\cdot \frac{1}{f}\;,$$

where *K* is Boltzmann’s constant, ∆f is the frequency range, and $K_{f}$ is a process-dependent parameter for 1/f noise. For α≪1 and a large OTA DC gain, the thermal noise of a large $R_{M1}$ and the 1/f noise are the main components of the noise source for the CS integrator. Therefore, the input equivalent noise of the CS integrator is approximately given by:

(15) $$\bar{V_{n,in}}\;\approx \;\frac{\frac{1}{R_{M1}C_{0}s}\cdot \left[{\left(\bar{V_{n,in,OTA}^{2}}\right)}^{1/2}+{\left(\bar{V_{n,M1}^{2}}\right)}^{1/2}\right]}{\frac{R_{M2}}{R_{M1}R_{0}C_{0}s}},$$

where $\bar{V_{n,in,OTA}^{2}}$ is the input noise power of the OTA. Equation (15) can be rewritten as:

(16) $$\bar{V_{n,in}}\;\approx \;\frac{R_{0}}{R_{M2}}\cdot \left[{\left(\bar{V_{n,in,OTA}^{2}}\right)}^{1/2}+{\left(\bar{V_{n,M1}^{2}}\right)}^{1/2}\right].$$

Equation (16) suggests that the noise performance of the CS integrator can be optimized by the OTA and the value of $R_{0}/R_{M2}$. In practical implementation, $R_{M2}$ is kept constant by a constant bias voltage ($V_{DD}$) and $R_{0}$ is chosen to be a slightly smaller value to ensure that the input-referred noise is kept at a low level. Moreover, the noise performance of the OTA is enhanced through design techniques, as described in the last subsection.

### 4.4. Linear Frequency Tuning

As shown in Figure 3, the transistors $M_{1}$ and $M_{2}$ in the α block function as two voltage-control resistors with resistance values changing according to the overdrive voltage. In other words, the value α can be adjusted by the control voltages $V_{b1}$ and $V_{b2}$, which in turn, change the filter cutoff frequency. 

In this design, $V_{b2}$ is set to the supply voltage $V_{DD}$. Substituting α into Equation (2) or Equation (4), and using Equation (7), the cutoff frequency is given by:

(17) $$f_{c,NF/LPF}\approx \frac{1}{{2\pi R}_{0}C_{0}}\cdot \frac{{(W/L)}_{1}{(V}_{b1}\;{-\;V}_{ref}\;{-\;V}_{thn})}{{(W/L)}_{2}{(V}_{DD}\;{-\;V}_{ref}\;{-\;V}_{thn})}.$$

Equation (17) suggests that the cutoff frequency of the reconfigurable filter is proportional to $V_{b1}$ if the other variables are kept unchanged. Since the relationship between $\omega_{0}$ and $V_{b1}$ is approximately linear, the cutoff frequency for the NF and LPF can be tuned through the α block via appropriate values of $V_{b1}$. Although the cutoff frequencies in conventional designs are rather sensitive to the process used, these adverse effects can be compensated for in the proposed design by adjusting the gate voltage $V_{b1}$ at the expense of requiring a more accurate control voltage for tuning. 

## 5. Results

**Figure 9:**
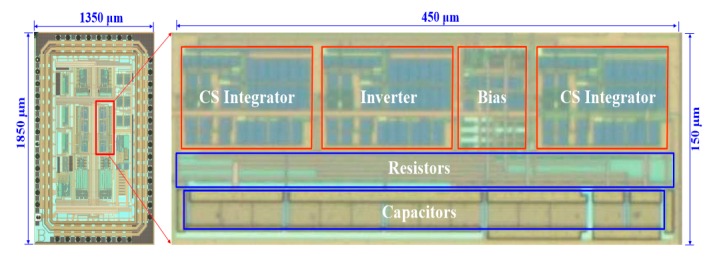
Microphotograph of the proposed second-order reconfigurable filter.

The proposed reconfigurable filter was fabricated using a SMIC 0.18 μm CMOS process with supporting circuits, including a bias block. The bias circuit was implemented to offer the bias current and bias voltage to the filter. Figure 9 shows the die microphotograph of the proposed filter, where the core size was about 450 μm × 150 μm. Polyresistance and metal–insulator–metal (MIM) capacitance were employed in the CMOS process. With the proposed methodology, the resistors and capacitors used could be chosen at the kilo-ohm and picofarad levels, respectively, to achieve a small area.

### 5.1. Tunable Frequency Response

**Figure 10:**
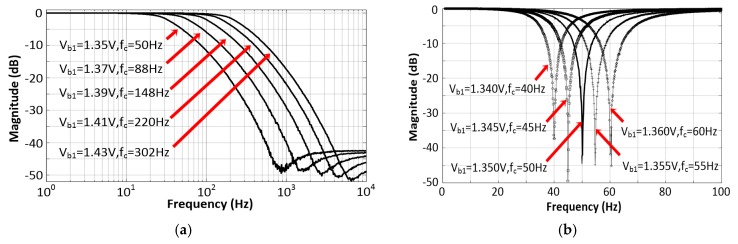
The measured tunable frequency responses of the proposed filter: (**a**) LPF and (**b**) NF.

The measured frequency responses of the proposed filter in two different configurations, namely NF and LPF, are shown in Figure 10a,b, respectively. The results demonstrate that impedance-boosting could create a sharp notch for the NF and a low cutoff for the LPF, with programmable frequencies. The NF and LPF had a relatively smooth pass-band and the cutoff frequency of the NF and LPF could be effectively tuned via $V_{b1}$. The NF provided approximately 45 dB of attenuation over a bandwidth of 10 Hz centered at 50 Hz without any obvious change of ripple under a 1.35 V bias. Figure 10b shows the response of the LPF in which a stop-band attenuation of 50 dB was achieved. The cutoff frequencies of the two configurations could be linearly programmed to various values through the voltage bias.

**Figure 11:**
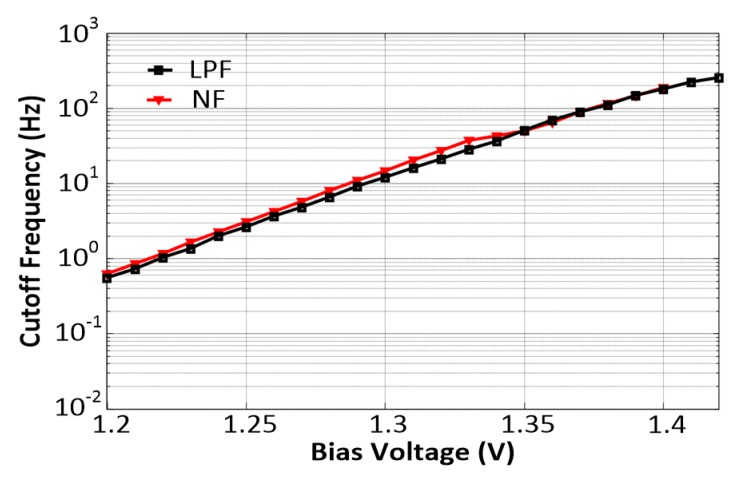
Tuning curve of cutoff the frequency when *V*_*b*1_ was varied from 1.2 V to 1.4 V.

Figure 11 depicts the cutoff frequency versus the bias voltage *V_b_*_1_ of the proposed reconfigurable filter. When *V_b_*_1_ varied from 1.2 to 1.4 V, the cutoff frequencies of the NF and LPF were seen to vary almost linearly from 60 mHz to 250 Hz and 50 mHz to 300 Hz, respectively.

### 5.2. Total Harmonic Distortion

**Figure 12:**
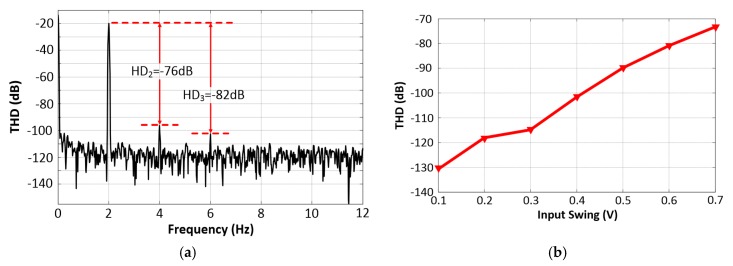
(**a**) Measured total-harmonic-distortion (THD) of the proposed filter with a 2 Hz, 200 mVpp sine wave input. (**b**) Measured THD of the proposed filter versus the input signal swing (2 Hz sine wave input).

Figure 12a depicts the measured THD of the proposed filter for a 200 mV_pp_ sine input at 2 Hz. As an example, measurements were taken with various bias voltage settings (1.39 V and 1.35 V), resulting in a 150 Hz corner frequency for the LPF and a 50 Hz central frequency for the NF configuration, respectively. The circuit outputted a very pure 2 Hz tone, showing no distortion above −96 dB, and it was bounded by the limited accuracy of the spectrum analyzer. The measured HD_2_ was around −76 dB (relative to the fundamental) at 4 Hz and the HD_3_ was −82 dB at 6 Hz, which demonstrates the high linearity achieved. 

The measured THD versus input signal swing is shown in Figure 12b. For this test, the amplitude of the input sine wave was changed while the frequency was kept at 2 Hz. It can be seen that the THD degraded as the input signal amplitude increased. The reason was that the dynamic range of the output changed with the input as the pass-band gain of our filter was set to one. As the drain-source voltage across the large variable MOS resistors increased, which resulted in a change in the equivalent impedance of the non-linear MOS resistors, degradation in the linearity was observed. Nonetheless, the measured results showed a low THD of −110 dB relative to the fundamental for a single tone of 2 Hz when the input signal amplitude was less than 350 mV. The linearity performance was well within the requirement of biopotential processing.

### 5.3. ECG Measurement

**Figure 13:**
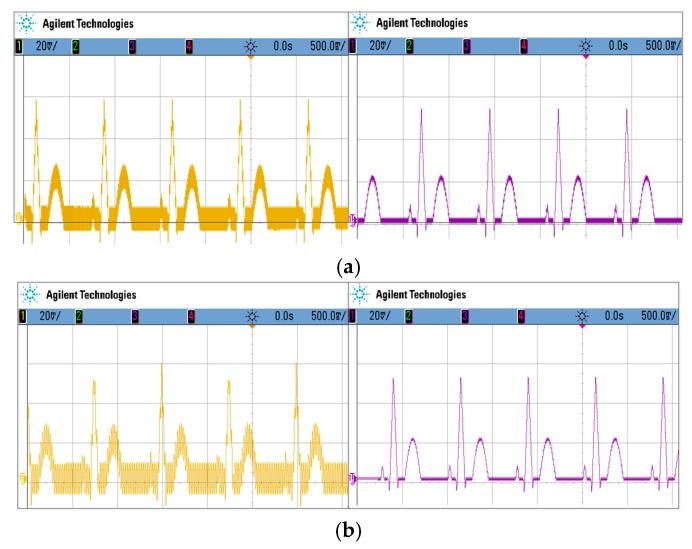
The simulated electrocardiograph (ECG) measurement results: (**a**) waveform captured without an LPF (left) and with an LPF (right), (**b**) waveform captured without an NF (left) and with an NF (right). *V_b1_* was set to 1.39 V for the LPF and 1.35 V for the NF. The input ECG signal was combined with (**a**) a 1 kHz, 10 mVpp and (**b**) a 50 Hz, 10 mVpp sine wave where the gain of the filter was about 0 dB.

To evaluate the suitability of the proposed filter in biopotential applications, the prototype filter was experimentally characterized with an amplified ECG signal using an off-the-shelf amplifier as input, where a simulated ECG was generated using the Fluke Medsim 300B (Fluke, WA, US). Figure 13a shows the measurement made under the LPF configuration at a bias of 1.39 V when the input ECG signal was mixed with a 1 kHz, 10 mVpp sine wave. It is evident that the high-frequency content was removed effectively. In Figure 13b, a 50 Hz, 10 mVpp sine wave emulating the effects of PLI was also added in the input of the filter. Under the NF configuration with a bias of 1.35 V, the waveform after filtering showed that the influence of PLI was significantly mitigated.

Table 1 shows a comparison of the proposed work with recently reported designs. The proposed filter had the best THD, smallest on-chip capacitor, and lowest input-referred noise relative to the conventional designs [6,16,17,26,27,28,29,30]. Moreover, the performance was achieved without scarifying the silicon area, except for that caused by a slight increase in power consumption. The capacitor/pole metrics were adopted such that similar filters with different orders could be meaningfully compared. As shown in the Table 1, the integrating capacitor per pole was 1.97 pF and 2.68 pF for the proposed LPF and NF, respectively, which was the smallest of all designs. It is noted that the proposed design had lower input-referred noise compared with other state-of-the-art filters. The main reason was that a large bias current and a high-gain OTA were employed to reduce the input-referred noise of the circuit. By employing a micro-amp-level bias current, the proposed filter consumed around 25 μW from a single 1.8 V power supply, which consumed a larger power compared with References [6,26,29] in exchange for better noise performance. As noise is a key parameter for biopotential signal conditioning due to their low voltage amplitude, it is crucial to achieve a good noise performance. The measurement results demonstrated that the proposed reconfigurable filter and design techniques are attractive approaches for realizing a tunable filter with a very low cutoff frequency and small-sized on-chip capacitors, and they are valuable for biopotential acquisition systems.

## 6. Conclusions

A reconfigurable continuous-time filter with a compact area and high linearity has been presented. The proposed filter has a flexible structure as it has a wide tunable bandwidth and can be used as either an LPF or an NF. It adopts a new OTA-based CS integrator to achieve a large time constant and biquad filter topology, as well as an improved analog inverter to obtain a low distortion. Consequently, a large time constant τ, good linearity, and low noise performance was achieved, while requiring a small silicon area. The proposed filter was fabricated in a standard 0.18 μm CMOS process and the chip size was around 450 μm × 150 μm. A comparison with conventional designs revealed that the proposed filter is very attractive in that it achieved the lowest harmonic distortion, smallest on-chip capacitance, and lowest input-referred noise among these designs. The proposed filter structure and design techniques serve as a good alternative to conventional continuous-time low-frequency filters. They are particularly valuable to low-frequency physiological signal filtering applications.

## Figures and Tables

**Table 1:** Summary and comparison of low-pass filters and notch filters for biomedical sensor applications.

	Low-Pass Filter					Notch Filter				
Parameter	2009 [17]	2018 [6]	2018 [26]	2019 [27] *	This Work	2005 [16]	2013 [28]	2013 [29]	2017 [30] *	This Work
Filter Order	1	4	4	4	2	5	8	2/4	5	2
Tuning Range (Hz)	0.002–90	100	100–300	0.03–100	0.05–300	30–67	10–1k	40–80	0–47.2	0.06–250
THD (dB)	−48.9 @50 Hz 100 mV	−50 @20 Hz 25 mV	−50 @20 Hz 25 mV	−40 @10 Hz	−76 @2 Hz 20 mV	−49.7 @8 Hz 50 mV	−34 @15 Hz 1 μA	−70/−65 @100 mV	−60.7 @10 Hz 30 mV	−76 @2 Hz 20 mV
Area (mm^2^)	0.07	0.11	0.1	0.737	0.0675	0.25	12.5	1	0.314	0.0675
Capacitor per pole (pF) ^$^	40	9.625	11.785	71.2	1.97	20	25	30.2	7.72	2.68
Pass-Band Gain (dB)	0	−0.05	−0.09	1.14	0	0.1	0	0	−2.08	0
Input-Referred Noise (μVrms)	32	80.5	39.38	129.2	5.5 ^&^	243	NA	NA	170.3	6.4 ^&^
Supply (V)	1	0.9	1.5	1.8	1.8	3	2	1.5	1.2	1.8
Process (μm)	0.35	0.35	0.35	0.18	0.18	0.35	0.35	0.18	0.13	0.18

* Simulation results; ^$^ Calculated by the authors; ^&^ The integration frequency range is from 0.05 Hz to 150 Hz.
